# Severe Acute Respiratory Syndrome Coronavirus 2 Antibody Response After Heterologous Immunizations With ChAdOx1/BNT162b2 in End-Stage Renal Disease Patients on Hemodialysis

**DOI:** 10.3389/fimmu.2022.894700

**Published:** 2022-06-06

**Authors:** Dae Kyu Kim, Su Woong Jung, Ju-Young Moon, Kyung Hwan Jeong, Hyeon Seok Hwang, Jin Sug Kim, Sang-Ho Lee, So-Young Kang, Yang Gyun Kim

**Affiliations:** ^1^Division of Nephrology, Department of Internal Medicine, Kyung Hee University, Seoul, South Korea; ^2^Department of Laboratory Medicine, College of Medicine, Kyung Hee University, Seoul, South Korea

**Keywords:** COVID-19, ChAdOx1, BNT162b2, hemodialysis, anti-RBD IgG, heterologous vaccination

## Abstract

The Korean government decided to schedule heterologous vaccinations on dialysis patients for early achievement of immunization against Coronavirus disease 2019(COVID-19). However, the effects of heterologous immunizations in hemodialysis (HD) patients are unclear. One hundred (HD) patients from Gangdong Kyung Hee University Hospital and Kyung Hee Medical Center and 100 hospital workers from Gangdong Kyung Hee University Hospital were enrolled in this study. The HD patients received the mixing schedule of ChAdOx1/BNT162b2 vaccinations at 10-week intervals, while hospital workers received two doses of ChAdOx1 vaccines at 12-week intervals. Serum IgG to a receptor-binding domain (RBD) of the S1 subunit of the spike protein of SARS-CoV-2 was measured 1 month after the first dose, 2 months and 4 months after the second dose. The median [interquartile range] anti-RBD IgG was 82.1[34.5, 176.6] AU/ml in HD patients and 197.1[124.0, 346.0] AU/ml in hospital workers (P < 0.001) after the first dose. The percentage of positive responses (IgG > 50 AU/ml) was 65.0% and 96.0% among the both group, respectively (P < 0.001). The anti-RBD IgG levels increased significantly by 2528.8 [1327.6, 5795.1] AU/ml with a 100.0% positive response rate in HD patients 2 months after the second dose, which was higher than those in hospital workers 981.4[581.5, 1891.4] AU/ml (P < 0.001). Moreover, anti-RBD IgG remains constantly high, and positive response remains 100% in HD patients 4 months after the second dose. This study suggests that heterologous vaccinations with ChAdOx1/BNT162b2 can be an alternative solution on HD patients for early and strong induction of humoral response.

## Introduction

Patients with end-stage renal disease (ESRD) are vulnerable to coronavirus disease 2019(COVID-19) caused by severe acute respiratory syndrome coronavirus 2(SARS-CoV-2). Reportedly, the mortality rate is 4–8 times higher than that in the general population (1–4). Therefore, vaccination against COVID-19 should be preferentially administered to ESRD patients (3). The Korean government approved the mixing of COVID-19 vaccines for the early achievement of immunization. However, the effectiveness of mixing COVID-19 vaccines in ESRD patients on dialysis has not been elucidated. This study investigated the humoral response following a heterologous vaccination schedule of ChAdOx1/BNT162b2 in ESRD patients receiving maintenance hemodialysis (HD).

## Materials and Methods

This study included 200 participants including 100 HD patients from two hospitals (Gangdong Kyung Hee University Hospital and Kyung Hee Medical Center) and 100 hospital workers from Gangdong Kyung Hee University Hospital. HD patients received the first dose of ChAdOx1 (April 23 - May 7, 2021) and their second dose of BNT162b2 (July 9 - July 22, 2021) at a 10-week interval. All hospital workers received two doses of ChAdOx1 vaccines at 12-week intervals (first: March 4 – March 10, 2021; second: May 21 – June 8, 2021). Serologic tests were performed 1 month after the first dose, and 2 months and 4 months after the second dose. Serum Immunoglobulin G to the receptor-binding domain of the S1 subunit of the spike protein of SARS-CoV-2 (anti-RBD IgG) was detected using the ARCHITECT IgG II Quant test (Abbott Laboratories). The Wuhan-Human 1 coronavirus (GenBank accession number MN908947) was used for the generation of anti-RBD IgG (5). This assay is an automated, two-step chemiluminescent microparticle immunoassay. The analytical measurement interval is stated as 21 to 40,000 AU/ml, and the titer over 50.0 AU/ml is defined as a positive result (6). Baseline clinical information was collected within a month before the first dose of vaccination in HD patients. This study was approved by the Clinical Institutional Review Board of Gangdong Kyung Hee University Hospital (KHNMC 2021-11-013). Data are expressed as means ± standard deviations (SD) or median [interquartile range (IQR)], and were compared using the Student’s t-test or the Wilcoxon rank-sum test. Comparison of decay rate 2 months after the second dose between groups was performed through nonparametric mixed models for exponential distribution. All *P* values were 2-tailed, and statistical significant value of *P* was considered as <0.05. The statistical analyses were performed using the SPSS software (version 22.0; SPSS, IBM Corp., Armonk, NY, USA) and R software (version 4.0.0).

## Results

**Figure 1:**
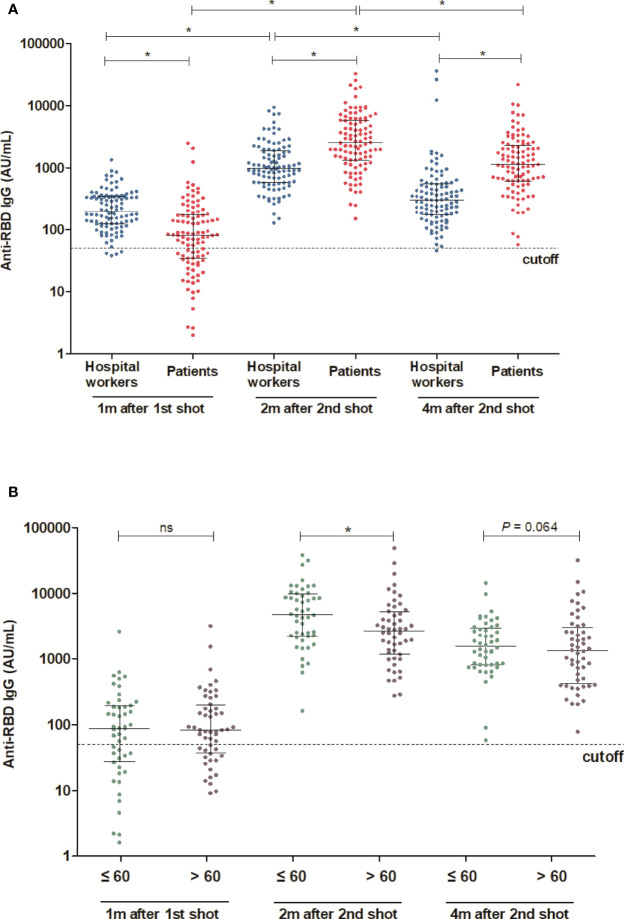
Comparison of antibody response between HD patients and hospital workers **(A)** Comparison of antibody levels following the first and second shot of vaccine between HD patients and hospital workers. **(B)** Comparison of antibody levels 2 month after the second shot of vaccine between younger and older HD patients (Age cut-off was 60 years). *P < 0.05, Dotted lines in **(A)** and **(B)** indicate the manufacturer’s pre-specified thresholds: anti-RBD IgG ≥ 50. Solid lines indicate median and interquartile range. m, months; w, weeks; ns, not significance.

**Figure 2:**
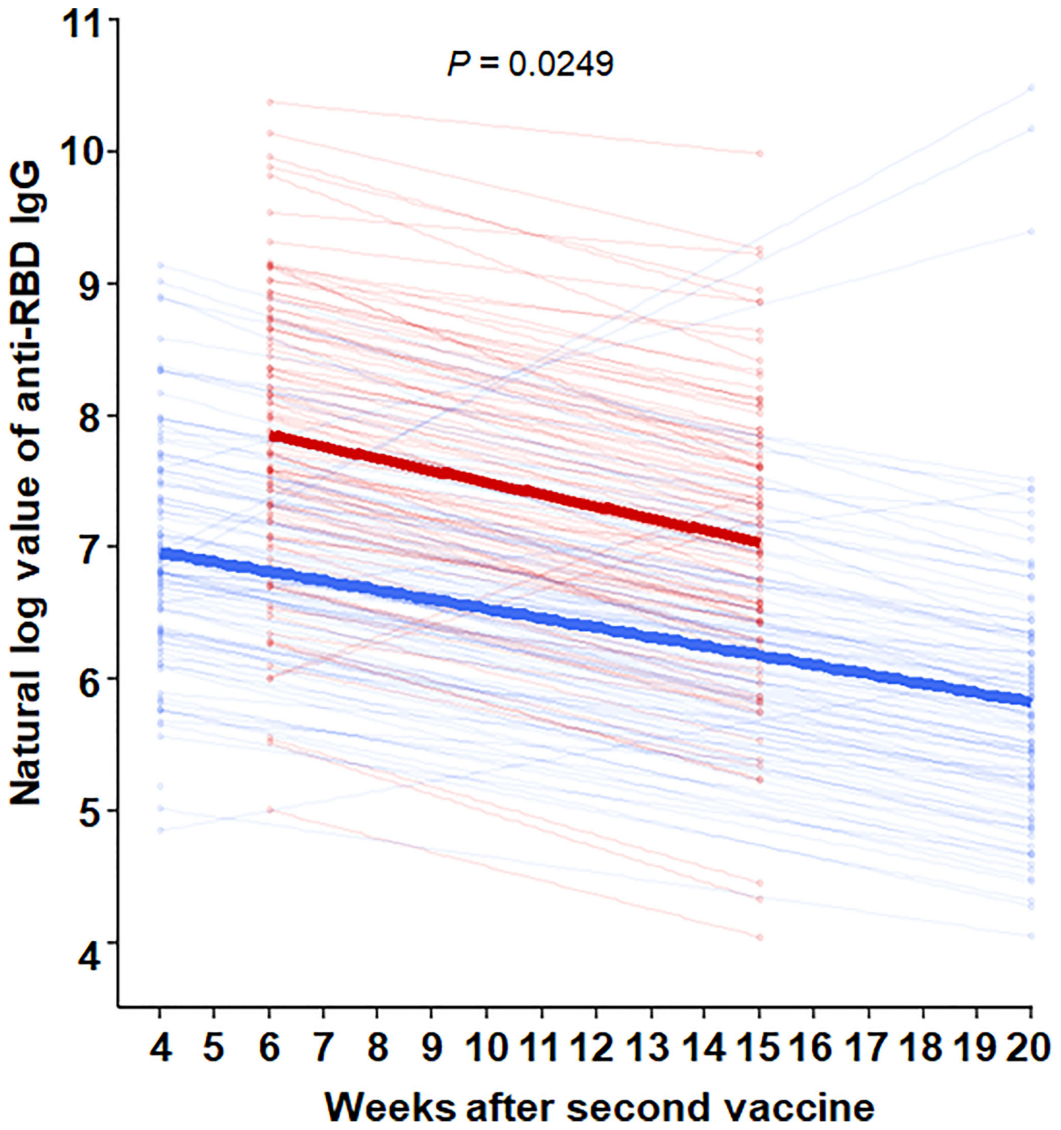
Comparison of decay rate of anti-RBD IgG after second dose of vaccine between HD patients and hospital workers through nonparametric mixed models for exponential distribution. Red line = HD patients, with formula [Anti-RBD IgG = e^(8.40-0.09*weeks after second vaccine)^]. Blue line = hospital workers, with the formula [Anti-RBD IgG = e^(8.40-0.07*weeks after second vaccine)^].

The mean age was 59.5 years in HD patients and 43.5 years in hospital workers (*P* < 0.001) (**Table 1**). The median [interquartile range] anti-RBD IgG level was 82.1[34.5, 176.6] AU/ml in HD patients and 197.1[124.0, 346.0] AU/ml in the hospital workers (*P* < 0.001) after the first dose of vaccine. The percentage of positive responses (IgG > 50 AU/ml) was 65.0% and 96.0% among the HD patients and hospital workers, respectively (*P* < 0.001). The anti-RBD IgG levels increased significantly by 2528.8[1327.6, 5795.1] AU/ml with a 100.0% positive response rate in HD patients and 981.4[581.5, 1891.4] with a 100.0% positive response rate in the hospital workers 2 months after the second dose of vaccine. Moreover, anti-RBD IgG remains constantly high as the level of 1143.7[619.9, 2337.1] AU/ml and a positive response was 100% in HD patients and 305.6[177.1, 550.2] AU/ml and a positive response rate was 99% in the hospital workers 4 months after the second dose of vaccine (**Figure 1A**). We divided the HD patients according to the age of 60. The antibody levels were augmented in the younger group compared with the older group (**Figure 1B**). The decay rate of anti-RBD IgG after second dose of vaccine is greater in HD patients (slope = -0.09) compared with hospital workers (slope = -0.07) (*P* = 0.0249) (**Figure 2**). Approximately 35.0% of the HD patients had mild symptoms (myalgia, fever, nausea, and pain at the injection site) after the first dose. Similar events were observed in 32.0% of the HD patients after the second dose. Most of the symptoms were well controlled with acetaminophen. We followed the patients and hospital workers after the study period. Until 5 months after the second antibody measurement, all 200 subjects had never been infected with COVID-19.

**Table 1:** Baseline characteristics and antibody responses in HD patients and hospital workers.

	HD patients (n = 100)	Hospital workers (n = 100)	*P*
Age (years)	59.5 ± 10.4	43.5 ± 6.5	<0.001
Sex (M/F)	61/39	33/67	<0.001
BMI (kg/m^2^)	23.2 ± 4.7		
Charlson comorbidity index	5.3 ± 2.0		
Dialysis duration (Mo)	78.9 ± 53.6		
DM ESRD (%)	42(42)		
Immunosuppressant (%)	3(3)		
Hb (g/dL)	10.8 ± 1.0		
Albumin (g/dL)	4.0 ± 0.8		
Total cholesterol (mg/dL)	131.1 ± 30.7		
hsCRP (mg/L)	1.7 ± 5.9		
Kt/V	1.7 ± 0.3		
Intact PTH (pmol/L)	399.7 ± 351.5		
HbA1c (%)	6.2 ± 1.2		
Anti-RBD IgG 1m after 1^st^ shot (AU/mL)	82.1 [34.5, 176.6]	177.4 [89.8, 347.2]	<0.001
Positive rate 1m after 1^st^ shot (%)	65.0	96%	<0.001
Anti-RBD IgG 2m after 2^nd^ shot (AU/mL)	2528.8[1327.6,5795.1]	981.4[581.5, 1891.4]	<0.001
Positive rate 2m after 2^nd^ shot (%)	100.0	100.0	–
Anti-RBD IgG 4m after 2^nd^ shot (AU/mL)	1143.7[619.9,2337.1]	305.6[177.1, 550.2]	<0.001
Positive rate 4m after 2^nd^ shot (%)	100.0	99.0	0.500

## Discussion

The humoral response after COVID-19 vaccination was slightly decreased in dialysis patients compared with healthy controls (7, 8). The decreased generation of antibodies might be associated with elevated SARS-CoV-2 infection rate even after the achievement of complete vaccination (9). Our study showed a similar finding that both anti-RBD IgG titer and positive rate were lower in HD patients than in hospital workers after the first dose of vaccination. Uremic toxins are thought as the reason for the lower humoral response in patients undergoing HD (10). Mixing vaccines can be an attractive solution to strengthen immunization and mitigate the vaccine shortage. Due to the vaccine mismatch between supply and demand, Korean government decided to schedule COVID-19 vaccination for almost medical staff as ChAdOx1/ChAdOx1 at 12-week intervals. For the ESRD patients classified by the high-risk group, ChAdOx1/BNT162b2 combination at 10-week intervals was allotted to achieve an earlier accomplishment of vaccine completion. Several studies have identified that heterologous ChAdOx1/BNT162b2 vaccines generated higher antibody levels, neutralizing capacity, and T cell reactivity than homologous ChAdOx1 or BNT162b2 vaccines (11). This study revealed that the humoral response elicited by heterologous vaccines in HD patients well remained 4 months after the second vaccine and was still higher than that in hospital workers. This study might be the first report to show the efficacy of heterologous vaccines in ESRD patients on HD. The seroconversion rate following homologous vaccines in ESRD patients was lower (average 87.3%, 70.5-98.0%) than in healthy controls (average 99.5%, 95.1-100.0%) (8). However, our study showed 100% of seroconversion in ESRD patients after 2 shots of heterologous vaccines, which is better efficacy than homologous vaccines. However, the decrease of anti-RBD IgG per week after the second vaccine was faster in HD patients than in hospital workers, which suggests a third booster shot is needed. In line with us, the antibodies following the two doses of BNT162b2 was rapidly declined in dialysis patients compared with those of general populations (12). Recent evidence has already proven the need for a third vaccine dose since neutralizing antibodies decreases rapidly 5 months after the second shot (13). A large observational study has proven that the third BNT162b2 shot decreased the risk of COVID-19 and serious complications following COVID-19 (14). Moreover, the third dose of the BNT162b2 vaccine substantially increased antibody levels in dialysis patients (15). Even though several countries have already used heterologous vaccines, the evidence regarding strong immunization and safety was scarce. Our results identified that the vaccine-related adverse effects were non-severe and well-tolerated. Patients previously infected with SARS-CoV-2 showed augmented antibody response following a single dose of COVID-19 vaccine compared with SARS-CoV-2-naïve patients (16, 17). This strong humoral response in previously infected persons might be caused by the formation of a robust cellular response after infection. Consistent with this finding, mRNA vaccines may easily boost humoral responses in the basement of primed cellular immunity *via* vector vaccines (18). Increased immunization with a mixture of vaccines has already been identified in other vaccines. Mice experiment demonstrated that an alternative vaccine consisting of an mRNA and an adenovirus vaccine against SARS-CoV-2 induced superior humoral as well as cellular response (19). Our study have several limitations. We did not measure neutralizing antibodies against SARS-CoV-2. Several studies already revealed that the anti-RBD IgG was highly compatible with the result of the plaque reduction neutralization test for the reference virus (SARS-CoV-2 FFM1 isolate) (20, 21). However, there are no available results to clarify the association between anti-RBD IgG and recent Variant of Concern (VOC) including Delta and Omicron. It is necessary to examine whether serologic antibody tests synthesized from Wuhan-Human 1 coronavirus can reflect neutralizing antibodies of VOCs. Although the Abbott serologic quant test well detected the Alpha (B.1.1.7) and Beta (B.1.351) variants, it was not tested on the Gamma (P.1), Delta (B.1.617.2), and Omicron (B. 1.1.529). The serologic assays targeting the spike protein or RBD can present reduced sensitivity in detecting antibodies generated against the variants. Nevertheless, natural infection and vaccinations produced a polyclonal antibody response targeting several regions of the spike protein (22). Moreover, a recent study has shown that mRNA vaccine-induced spike protein-specific antibodies continue to drive Fc effector functions, suggesting a capacity for extra-neutralizing antibodies to contribute to disease control (23). Thus, it is likely that the current serologic testing could reflect the humoral response against COVID-19 well. This study suggests that heterologous vaccination with ChAdOx1/BNT162b2 can be an alternative solution on the HD patients for early and strong induction of humoral response.

## Data Availability Statement

The raw data supporting the conclusions of this article will be made available by the authors, without undue reservation.

## Ethics Statement

The studies involving human participants were reviewed and approved by the Clinical Institutional Review Board of Gangdong Kyung Hee University Hospital (KHNMC 2021-11-013). The patients/participants provided their written informed consent to participate in this study.

## Author Contributions

YGK and SYK conceived the research questions and designed the analysis. SWJ, KHJ, HSH, JSK, SHL, and J-YM conducted the data collection. DKK and YGK drafted the manuscript. All authors reviewed the results and commented on the manuscript. All authors read and approved the final manuscript.

## Funding

This work was supported by a grant from Kyung Hee University in 2020 (KHU-20201225).

## Conflict of Interest

The authors declare that the research was conducted in the absence of any commercial or financial relationships that could be construed as a potential conflict of interest.

## Publisher’s Note

All claims expressed in this article are solely those of the authors and do not necessarily represent those of their affiliated organizations, or those of the publisher, the editors and the reviewers. Any product that may be evaluated in this article, or claim that may be made by its manufacturer, is not guaranteed or endorsed by the publisher.

## References

[1] Couchoud C Bayer F Ayav C Bechade C Brunet P Chantrel F. Low Incidence of Sars-Cov-2, Risk Factors of Mortality and the Course of Illness in the French National Cohort of Dialysis Patients. Kidney Int (2020) 98(6):1519–29. doi: 10.1016/j.kint.2020.07.042 PMC7445552 32858081

[2] Hsu CM Weiner DE. Covid-19 in Dialysis Patients: Outlasting and Outsmarting a Pandemic. Kidney Int (2020) 98(6):1402–4. doi: 10.1016/j.kint.2020.10.005 PMC7552964 33065131

[3] Turgutalp K Ozturk S Arici M Eren N Gorgulu N Islam M. Determinants of Mortality in a Large Group of Hemodialysis Patients Hospitalized for Covid-19. BMC Nephrol (2021) 22(1):29. doi: 10.1186/s12882-021-02233-0 33446135 PMC7808398

[4] Valeri AM Robbins-Juarez SY Stevens JS Ahn W Rao MK Radhakrishnan J. Presentation and Outcomes of Patients With Eskd and Covid-19. J Am Soc Nephrol (2020) 31(7):1409–15. doi: 10.1681/ASN.2020040470 PMC7350989 32467113

[5] English E Cook LE Piec I Dervisevic S Fraser WD John WG. Performance of the Abbott Sars-Cov-2 Igg Ii Quantitative Antibody Assay Including the New Variants of Concern, Voc 202012/V1 (United Kingdom) and Voc 202012/V2 (South Africa), and First Steps Towards Global Harmonization of Covid-19 Antibody Methods. J Clin Microbiol (2021) 59(9):e0028821. doi: 10.1128/JCM.00288-21 34260272 PMC8373017

[6] Abbott. Abbott ARCHITECT SARS-CoV-2 IgG II Quant Reagent Instructions for Use (2020). Available at: https://www.corelaboratory.abbott/int/en/offerings/segments/infectious-disease/sars-cov-2 (Accessed December 2, 2020).

[7] Benotmane I Gautier-Vargas G Cognard N Olagne J Heibel F Braun-Parvez L. Weak Anti-Sars-Cov-2 Antibody Response After the First Injection of an Mrna Covid-19 Vaccine in Kidney Transplant Recipients. Kidney Int (2021) 99(6):1487–9. doi: 10.1016/j.kint.2021.03.014 PMC7997264 33775674

[8] Chen JJ Lee TH Tian YC Lee CC Fan PC Chang CH. Immunogenicity Rates After Sars-Cov-2 Vaccination in People With End-Stage Kidney Disease: A Systematic Review and Meta-Analysis. JAMA Netw Open (2021) 4(10):e2131749. doi: 10.1001/jamanetworkopen.2021.31749 34709385 PMC8554642

[9] Yanay NB Freiman S Shapira M Wishahi S Hamze M Elhaj M. Experience With Sars-Cov-2 Bnt162b2 Mrna Vaccine in Dialysis Patients. Kidney Int (2021) 99(6):1496–8. doi: 10.1016/j.kint.2021.04.006 PMC8055922 33887318

[10] Danthu C Hantz S Dahlem A Duval M Ba B Guibbert M. Humoral Response After Sars-Cov-2 Mrna Vaccination in a Cohort of Hemodialysis Patients and Kidney Transplant Recipients. J Am Soc Nephrol (2021) 32(9):2153–8. doi: 10.1681/ASN.2021040490 PMC8729854 34135083

[11] Glenn DA Hegde A Kotzen E Walter EB Kshirsagar AV Falk R. Systematic Review of Safety and Efficacy of Covid-19 Vaccines in Patients With Kidney Disease. Kidney Int Rep (2021) 6(5):1407–10. doi: 10.1016/j.ekir.2021.02.011 PMC7870446 33585728

[12] Berar-Yanay N Freiman S Shapira M Saffoury A Elemy A Hamze M. Waning Humoral Response 3 to 6 Months After Vaccination With the Sars-Cov-2 Bnt162b2 Mrna Vaccine in Dialysis Patients. J Clin Med (2021) 11(1):64. doi: 10.3390/jcm11010064 35011801 PMC8745040

[13] Falsey AR Frenck RW Jr. Walsh EE Kitchin N Absalon J Gurtman A. Sars-Cov-2 Neutralization With Bnt162b2 Vaccine Dose 3. N Engl J Med (2021) 385(17):1627–9. doi: 10.1056/NEJMc2113468 PMC8461567 34525276

[14] Barda N Dagan N Cohen C Hernan MA Lipsitch M Kohane IS. Effectiveness of a Third Dose of the Bnt162b2 Mrna Covid-19 Vaccine for Preventing Severe Outcomes in Israel: An Observational Study. Lancet (2021) 398(10316):2093–100. doi: 10.1016/S0140-6736(21)02249-2 PMC8555967 34756184

[15] Bensouna I Caudwell V Kubab S Acquaviva S Pardon A Vittoz N. Sars-Cov-2 Antibody Response After a Third Dose of the Bnt162b2 Vaccine in Patients Receiving Maintenance Hemodialysis or Peritoneal Dialysis. Am J Kidney Dis (2022) 79(2):185–92 e1. doi: 10.1053/j.ajkd.2021.08.005 34508833 PMC8425695

[16] Krammer F Srivastava K Alshammary H Amoako AA Awawda MH Beach KF. Antibody Responses in Seropositive Persons After a Single Dose of Sars-Cov-2 Mrna Vaccine. N Engl J Med (2021) 384(14):1372–4. doi: 10.1056/NEJMc2101667 PMC8008743 33691060

[17] Prendecki M Clarke C Brown J Cox A Gleeson S Guckian M. Effect of Previous Sars-Cov-2 Infection on Humoral and T-Cell Responses to Single-Dose Bnt162b2 Vaccine. Lancet (2021) 397(10280):1178–81. doi: 10.1016/S0140-6736(21)00502-X PMC7993933 33640037

[18] Pollard AJ Launay O Lelievre JD Lacabaratz C Grande S Goldstein N. Safety and Immunogenicity of a Two-Dose Heterologous Ad26.Zebov and Mva-Bn-Filo Ebola Vaccine Regimen in Adults in Europe (Ebovac2): A Randomised, Observer-Blind, Participant-Blind, Placebo-Controlled, Phase 2 Trial. Lancet Infect Dis (2021) 21(4):493–506. doi: 10.1016/S1473-3099(20)30476-X 33217361

[19] Spencer AJ McKay PF Belij-Rammerstorfer S Ulaszewska M Bissett CD Hu K. Heterologous Vaccination Regimens With Self-Amplifying Rna and Adenoviral Covid Vaccines Induce Robust Immune Responses in Mice. Nat Commun (2021) 12(1):2893. doi: 10.1038/s41467-021-23173-1 34001897 PMC8129084

[20] Kohmer N Ruhl C Ciesek S Rabenau HF. Utility of Different Surrogate Enzyme-Linked Immunosorbent Assays (Selisas) for Detection of Sars-Cov-2 Neutralizing Antibodies. J Clin Med (2021) 10(10):2128. doi: 10.3390/jcm10102128 34069088 PMC8157164

[21] Schmidt F Weisblum Y Rutkowska M Poston D DaSilva J Zhang F. High Genetic Barrier to Sars-Cov-2 Polyclonal Neutralizing Antibody Escape. Nature (2021) 600(7889):512–6. doi: 10.1038/s41586-021-04005-0 PMC9241107 34544114

[22] Bartsch YC Tong X Kang J Avendano MJ Serrano EF Garcia-Salum T. Omicron Variant Spike-Specific Antibody Binding and Fc Activity Are Preserved in Recipients of Mrna or Inactivated Covid-19 Vaccines. Sci Transl Med (2022) 14(642):eabn9243. doi: 10.1126/scitranslmed.abn9243 35289637 PMC8995028

[23] Phelan T Dunne J Conlon N Cheallaigh CN Abbott WM Faba-Rodriguez R. Dynamic Assay for Profiling Anti-Sars-Cov-2 Antibodies and Their Ace2/Spike Rbd Neutralization Capacity. Viruses (2021) 13(7):1371. doi: 10.3390/v13071371 34372581 PMC8309970

